# SGLT1 is required for the survival of triple‐negative breast cancer cells via potentiation of EGFR activity

**DOI:** 10.1002/1878-0261.12530

**Published:** 2019-06-14

**Authors:** Huiquan Liu, Ayse Ertay, Ping Peng, Juanjuan Li, Dian Liu, Hua Xiong, Yanmei Zou, Hong Qiu, David Hancock, Xianglin Yuan, Wei‐Chien Huang, Rob M. Ewing, Julian Downward, Yihua Wang

**Affiliations:** ^1^ Department of Oncology, Tongji Hospital, Tongji Medical College Huazhong University of Science and Technology Wuhan China; ^2^ Biological Sciences, Faculty of Environmental and Life Sciences University of Southampton UK; ^3^ Oncogene Biology The Francis Crick Institute London UK; ^4^ Graduate Institute of Biomedical Sciences China Medical University Taichung Taiwan; ^5^ Center for Molecular Medicine China Medical University and Hospital Taichung Taiwan; ^6^ Department of Biotechnology, College of Health Science Asia University Taichung Taiwan; ^7^ Institute for Life Sciences University of Southampton UK

**Keywords:** EGFR, SGLT1, triple‐negative breast cancer

## Abstract

Sodium/glucose cotransporter 1 (SGLT1), an essential active glucose transport protein that helps maintain high intracellular glucose levels, was previously shown to interact with epidermal growth factor receptor (EGFR); the SGLT1–EGFR interaction maintains intracellular glucose levels to promote survival of cancer cells. Here, we explore the role of SGLT1 in triple‐negative breast cancer (TNBC), which is the most aggressive type of breast cancer. We performed TCGA analysis coupled to *in vitro* experiments in TNBC cell lines as well as *in vivo* xenografts established in the mammary fat pad of female nude mice. Tissue microarrays of TNBC patients with information of clinical–pathological parameters were also used to investigate the expression and function of SGLT1 in TNBC. We show that high levels of SGLT1 are associated with greater tumour size in TNBC. Knockdown of SGLT1 compromises cell growth *in vitro* and *in vivo*. We further demonstrate that SGLT1 depletion results in decreased levels of phospho‐EGFR, and as a result, the activity of downstream signalling pathways (such as AKT and ERK) is inhibited. Hence, targeting SGLT1 itself or the EGFR–SGLT1 interaction may provide novel therapeutics against TNBC.

**Abbreviations**
ATCC: American Type Culture Collection
DAVID: database for annotation, visualization and integration discovery
EGFR: epidermal growth factor receptor
HER2: human epidermal growth factor 2
IgG: immunoglobulin G
MTT: 3‐(4,5‐dimethyl‐thiazol‐2‐yl)‐2,5‐diphenyltetrazolium bromide
PD‐L1: programmed death‐ligand 1
SGLT1: sodium/glucose cotransporter 1
shRNA: short hairpin RNA
siRNA: short interfering RNA
SPF: specific pathogen‐free
TNBC: triple‐negative breast cancer

## 1 Introduction

Breast cancer is the most frequent cancer type in women with 1.38 million new cases (23%) each year throughout the world. It is the fifth leading cause of cancer death (458 000 deaths worldwide) and, globally, the most frequent female cancer deaths in developing and developed countries (Ferlay *et al.*, 2010). Triple‐negative breast cancer (TNBC), which lacks expressions of oestrogen, progesterone and human epidermal growth factor 2 (HER2) receptors, accounts for 10‐20% breast cancers with unsatisfactory therapeutic efficacy (O'Reilly *et al.*, 2015; Reis‐Filho and Tutt, 2008). TNBC is the most aggressive, high‐grade breast cancer type with high risk of metastasis and poor survival rate compared with the other breast cancer subtypes. Chemotherapy has been the only accepted systemic treatment option for TNBC for several years to increase the overall survival rate (Khosravi‐Shahi *et al.*, 2018). However, drug resistance occurs due to its heterogeneity (Du *et al.*, 2015), and this contributes to the recurrence of the metastatic disease (Lee and Djamgoz, 2018). Therefore, it is crucial to identify targeted therapies for TNBC.

Novel targeted therapies, such as endocrine therapies and targeting HER2, were discovered by mapping the genomic landscapes of breast cancer tumours. Genomic analysis has led to insights into the classification of TNBC such as the separation of TNBC into different subtypes based on gene expression (Criscitiello *et al.*, 2012; Garrido‐Castro *et al.*, 2019; Network, 2012). However, due to its complexity and heterogeneity, it has been challenging to identify targeted therapies for TNBC (Lehmann *et al.*, 2011). Atezolizumab (TECENTRIQ^®^, Roche, Basel, Switzerland), an antiprogrammed death‐ligand 1 (PD‐L1) monoclonal antibody, was approved recently as a first targeted therapy for TNBC with combination of chemotherapy (Abraxane; nab^®^‐Paclitaxel, Summit, NJ, USA) (Cyprian *et al.*, 2019). As atezolizumab plus chemotherapy combination is only successful for PD‐L1 positive TNBC and TNBC is a heterogeneous disease, further molecular studies are required to identify novel therapeutic targets for TNBC. Over the past decades, potential targeted therapies were discovered for TNBC by targeting different signalling pathways such as epidermal growth factor receptor (EGFR) (Crozier *et al.*, 2016; Nabholtz *et al.*, 2016; Schuler *et al.*, 2012; Shao *et al.*, 2017; Trédan *et al.*, 2015). However, targeting EGFR pathway was not very successful in part due to the resistance mechanisms or activation of different signalling pathway(s) (Baselga *et al.*, 2005; Baselga *et al.*, 2013; Carey *et al.*, 2012; Spector *et al.*, 2005). A previous study showed that inhibition of sodium/glucose cotransporter 1 (SGLT1) sensitized prostate cancer cells to EGFR inhibitors (gefitinib and erlotinib) (Wright *et al.*, 2011), although the precise mechanisms have not been elucidated. Knowing that SGLT1 mainly interacts with the autophosphorylation domain of EGFR (Ren *et al.*, 2013), we hypothesized that SGLT1 may regulate EGFR activity in TNBC.

Sodium/glucose cotransporter 1, encoded by the *SLC5A1* gene in humans, is an active glucose transporter, which utilizes sodium gradients to transport glucose into cells independent of extracellular glucose concentration (Rieg and Vallon, 2018; Wright *et al.*, 2011). Various studies have discovered that SGLT1 is overexpressed in different cancer types: prostate cancer (Blessing *et al.*, 2012), ovarian carcinoma (Lai *et al.*, 2012), oral squamous cell carcinoma (Hanabata *et al.*, 2012), head and neck carcinoma (Wright *et al.*, 2011), pancreatic cancer (Casneuf *et al.*, 2008) and colorectal cancer (Guo *et al.*, 2011). In ovarian carcinoma, tumour development and poor prognosis of the disease is associated with the overexpression of SGLT1 (Lai *et al.*, 2012). Overexpression of SGLT1 also has linked with higher clinical stages of colorectal cancer (Guo *et al.*, 2011). Despite of all these observations, the role of SGLT1 in TNBC was not known. In this study, we report that SGLT1 is essential for the survival of TNBC cells *in vitro* and *in vivo*. This is achieved, at least in part, via potentiating EGFR activity.

## 2 Materials and methods

### 2.1 Cell culture and transfection

BT549, MDA‐MB‐468 and MDA‐MB‐436 cell lines, which all belong to triple‐negative breast carcinoma cells, were purchased from Cell Bank of the Institute of Basic medicine, Chinese Academy of Medical Sciences (Beijing, China), or obtained as NCI‐ICBP45 kit procured through American Type Culture Collection (ATCC; ATCC Breast Cancer Cell Panel, Manassas, VA, USA). BT549 cells were cultured in RPMI 1640 medium (Boster, Wuhan, China) supplemented with 10% FBS (Gibco, Carlsbad, USA), 1% antibiotics and 0.023 IU·mL^−1^ bovine insulin (Sigma‐Aldrich, Saint Louis, MO, USA). MDA‐MB‐436 and MDA‐MB‐468 cells were cultured in Dulbecco's modified Eagle's medium (Boster) with 20% or 10% FBS (Gibco) and 1% antibiotics. All cells were kept at 37 °C and 5% CO_2_. The short hairpin RNA (shRNA) product of SGLT1 (sh‐SGLT1) with a targeted sequence of ATCTTTCTCTTATTGGCAA and its negative control (sh‐NC) were purchased from GeneChem (Shanghai, China) and transfected into cells according to manufacturer’s instructions. Short interfering RNA (siRNA) oligos against SGLT1 (MU‐007589‐01‐0002) were purchased from Dharmacon (Lafayette, CO, USA). Sequences are available from Dharmacon or upon request. As a negative control, we used siGENOME RISC‐Free siRNA (Dharmacon). Cells were transfected with the indicated siRNA oligos at a final concentration of 35 nm using Dharmafect 2 reagent (Dharmacon).

### 2.2 Cell viability assay

Short hairpin RNA‐transfected cells were seeded into 96‐well plates with a density of 1 × 10^3^ cells per well and allowed to grow for 24, 48 and 72 h. When indicated time arrived, 3‐(4,5‐Dimethyl‐thiazol‐2‐yl)‐2,5‐diphenyltetrazolium bromide (MTT) solution (Sangon Biotech, Shanghai, China) was added into the medium with a final concentration of 0.5 mg·mL^−1^. The  solution in each well was carefully sucked after incubation for 4 h at 37 °C, replaced by 100 µL formazan solubilization solution and gently mix for 10 min. OD values at 570 nm were measured using Microplate Reader (Bioteck Instrument, Winooski, VT, USA).

### 2.3 Western blot analysis

Western blot analysis was performed with lysates from cells or tissues with urea buffer (8 m urea, 1 m thiourea, 0.5% CHAPS, 50 mm DTT and 24 mm spermine). For immunoprecipitations, the cells were lysed for 30 min at 4 °C in pNAS buffer [50 mm Tris/HCl (pH 7.5), 120 mm NaCl, 1 mm EDTA and 0.1% Nonidet P‐40], with protease inhibitors. Indicated antibodies and immunoglobulin G (IgG) agarose were added to the lysate for 16 h at 4 °C. Immunoprecipitates were washed four times with cold PBS followed by the addition of SDS sample buffer. The bound proteins were separated on SDS polyacrylamide gels and subjected to immunoblotting with the indicated antibodies. Primary antibodies were from Abcam (Cambridge, UK) (SGLT1, 1 : 1000, ab14686; β‐actin, 1 : 2000, ab8227; β‐tubulin, 1 : 5000, ab6046; GAPDH, 1 : 2000, ab9385; Phospho‐EGFR^Tyr1068^, 1 : 1000, ab40815), Cell Signaling Technology (Leiden, Netherlands) [SGLT1, 1 : 1000, 5042; Phospho‐EGFR^Tyr1068^, 1 : 1000, 3777, D7A5; EGFR, 1 : 1000, 4267, D38B1; Phospho‐AKT Ser473, 1 : 1000, 9271; Phospho‐AKT Thr308, 1 : 1000, 4056, 244F9; AKT, 1 : 1000, 9272; Phospho‐ERK (Thr202/Tyr204), 1 : 1000, 9101; ERK, 1 : 1000, 9102; Cleaved PARP Asp214, 1 : 1000, 9541] and Millipore (Burlington, MA, USA) (PTEN, 1 : 1000, 04‐409). Signals were detected using an ECL detection system (GE Healthcare) (Chicago, IL, USA) or an Odyssey imaging system (LI‐COR), and evaluated by ImageJ 1.42q software (National Institutes of Health) (Berhesda, MD, USA).

### 2.4 Immunofluorescence microscopy

Cells were fixed in 4% PBS‐paraformaldehyde for 15 min, incubated in 0.1% Triton X‐100 for 5 min on ice, then incubated in 0.2% Fish Skin Gelatine in PBS for 1 h, and stained for 1 h with an anti‐Phospho‐EGFR^Tyr1068^ (1 : 100; Cell Signaling Technology, 3777). Protein expression was detected using Alexa Fluor (1 : 400; Molecular Probes, Eugene, OR, USA) for 20 min. TO‐PRO‐3 (Invitrogen, Waltham, MA, USA) was used to stain nucleic acids (1 : 2000).

### 2.5 Animal study

Animal experiments were carried out in accordance with the guidelines and approved protocols of the Ethics Committee of Tongji Hospital (Wuhan, China). Specific pathogen‐free (SPF) nude mice (female, 6 weeks) were purchased from Vital River Laboratory Animal Technology Co., Ltd. (Beijing, China) and housed under SPF condition. After adaptation for 1 week in condition of stable temperature and humidity with 12 h light–dark cycle, mice were randomly divided into sh‐NC group and sh‐SGLT1 group (*n* = 6 mice per group). ShRNA‐transfected MDA‐MB‐436 cells were harvested, rinsed and resuspended in PBS at a concentration of 2 × 10^8^ cells·mL^−1^, which were then placed on ice for subsequent operation. Orthotopic injection was performed in sterile condition in clean bench according to protocol described previously (Kocatürk and Versteeg, 2015) with adaptations. Briefly, after anesthetizing the mice intraperitoneally with sterilized 2% Avertin (Sigma‐Aldrich) with a dosage of 0.12 mL/10 g weight, mammary gland was exposed by making a small incision between the fourth nipple and the midline. A total of 1 × 10^7^ cells in 50 µL suspension were completely injected into mammary fat pad located in groin without leaking. Then, the incisions were sutured and mice were attended until they gain consciousness. The lengths and widths of xenografts were measured weekly by vernier calliper, and volume was calculated using following formula: *V* (mm^3^) = 1/2*length*widths^2^. At the end of experiment, mice were sacrificed, while xenografts were completely separated, measured and fixed by 4% paraformaldehyde for histological staining.

### 2.6 Immunohistochemical and H/E staining and scoring

Tissue microarray of TNBC patients with information of clinical–pathological parameters was purchased from Outdo Biotech (HBreD090Bc01; Shanghai, China). Paraffin‐embedded sections of xenograft tissues were subjected to deparaffinization and rehydration. H/E staining of sections was carried out using H/E staining kit (Beyotime, Shanghai, China) according to manufacturer’s instructions. For immunohistochemical staining of tissue microarray and sections of xenograft and antigen retrieval, blocking of non‐specific binding and incubation of primary antibodies at 4 °C overnight was sequentially conducted. The primary antibodies used were list as follows: anti‐phospho‐EGFR (ab40815; Abcam, 1 : 200) and anti‐SGLT1 (ab14686; Abcam, 1 : 100). After incubation with secondary goat anti‐rabbit immunoglobulin conjugated to peroxidase‐labelled dextran polymer (SV0002; Boster) at 37 °C for 1 h, visualization, counterstaining with haematoxylin and mounting were performed. Semiquantitative evaluations of protein expression were scored on the basis of the intensity and the percentage of phospho‐EGFR‐ or SGLT1‐positive tumour cells as previously described (Wang *et al.*, 2014).

### 2.7 TCGA data mining and pathway analysis

*SLC5A1*, mRNA expression z‐score (RNA Seq V2 RSEM) and molecular subtypes of breast invasive carcinoma (TCGA, Provisional) were obtained from cBioPortal for Cancer Genomics website (http://www.cbioportal.org). Molecular subtypes of breast samples were separated based on ER, PR and HER2 status, and mRNA expression of SGLT1 was analysed in each subtype in graphpad prism 8 (La Jolla, CA, USA) by ordinary one‐way ANOVA.

Two different data sets, TCGA_BRCA_RPPA‐2015‐02‐24 and TCGA_BRCA_exp_HiSeqV2‐2015‐02‐ 24, for RPPA protein expression and gene expression RNASeq (IlluminaHiSeq, San Diego, CA, USA), respectively, were extracted from UCSC Cancer Browser (https://genome-cancer.ucsc.edu/) for the analysis of genomic matrix data in TNBC samples. The data of TNBC samples that had SGLT1 mRNA expression from the cBioPortal for Cancer Genomics website were aligned with the samples in genomic matrix of TCGA data for both protein and gene expressions. For both protein and gene expression data sets, top 20% and bottom 20% of samples were chosen for high and low SGLT1 expression, respectively. Then, unpaired *t*‐test was performed to find the significantly different proteins/genes between high and low SGLT1 groups in r version 3.4.4 (Auckland, New Zealand), *P* < 0.05. For the protein expression of AKT_pT308 in TCGA protein data set, prism8 was used to plot scatter dot plot between high and low SGLT1 groups.

To explore the pathway analysis of the TCGA gene data analysis, the database for annotation, visualization and integration discovery (DAVID) functional annotation web tool was conducted (version 6.8; https://david.ncifcrf.gov (Huang *et al.*, 2009)). A total of 1325 genes, which were positively correlated with SGLT1 in TCGA gene data, were analysed with DAVID web tool and obtained the lists of enriched Kyoto Encyclopedia of Genes and Genomes pathway, REACTOME pathway, BIOCARTA, BBID and EC number. A *P* value ≤ 0.05 was considered significant. The pathways were sorted from lowest *P* value, and top 34 pathways were chosen which then were sorted with highest number of shared genes. Subsequently, we then plotted that histogram plot with the top 15 pathways in graphpad prism 8.

### 2.8 Statistical analysis

Comparison of two groups was statistically calculated by Student's *t*‐test. Chi‐square test or Fisher exact test were used to evaluate the relationship of SGLT1 expression and clinical parameters of TNBC. Correlation between expressions of SGLT1 and phospho‐EGFR was analysed using Pearson's correlation. Data were shown as mean ± SD. Statistical analysis was conducted using spss version 19.0 (Endicott, NY, USA). Unpaired *t*‐test was performed for TCGA data analysis in r version 3.4.4. To identify the statistical difference of AKT_pT308 between high and low SGLT1 group in TCGA protein data, unpaired *t*‐test was performed in graphpad prism 8 software.

## 3 Results

### 3.1 SGLT1 expression is higher in TNBC, and this associates with a larger tumour size

The expression of SGLT1 was upregulated in various cancer types (Blessing *et al.*, 2012; Casneuf *et al.*, 2008; Guo *et al.*, 2011; Hanabata *et al.*, 2012; Lai *et al.*, 2012; Wright *et al.*, 2011). To investigate whether the expression of SGLT1 is different between each molecular subtype of breast cancer, TCGA breast invasive carcinoma (Provisional) data were analysed. The mRNA levels of SGLT1 (*SLC5A*) were significantly higher in TNBC and HER2‐positive subtypes than luminal A and luminal B (Fig. S1). No significant difference between TNBC‐ and HER2‐positive subtypes was observed (Fig. S1).

**Figure 1:**
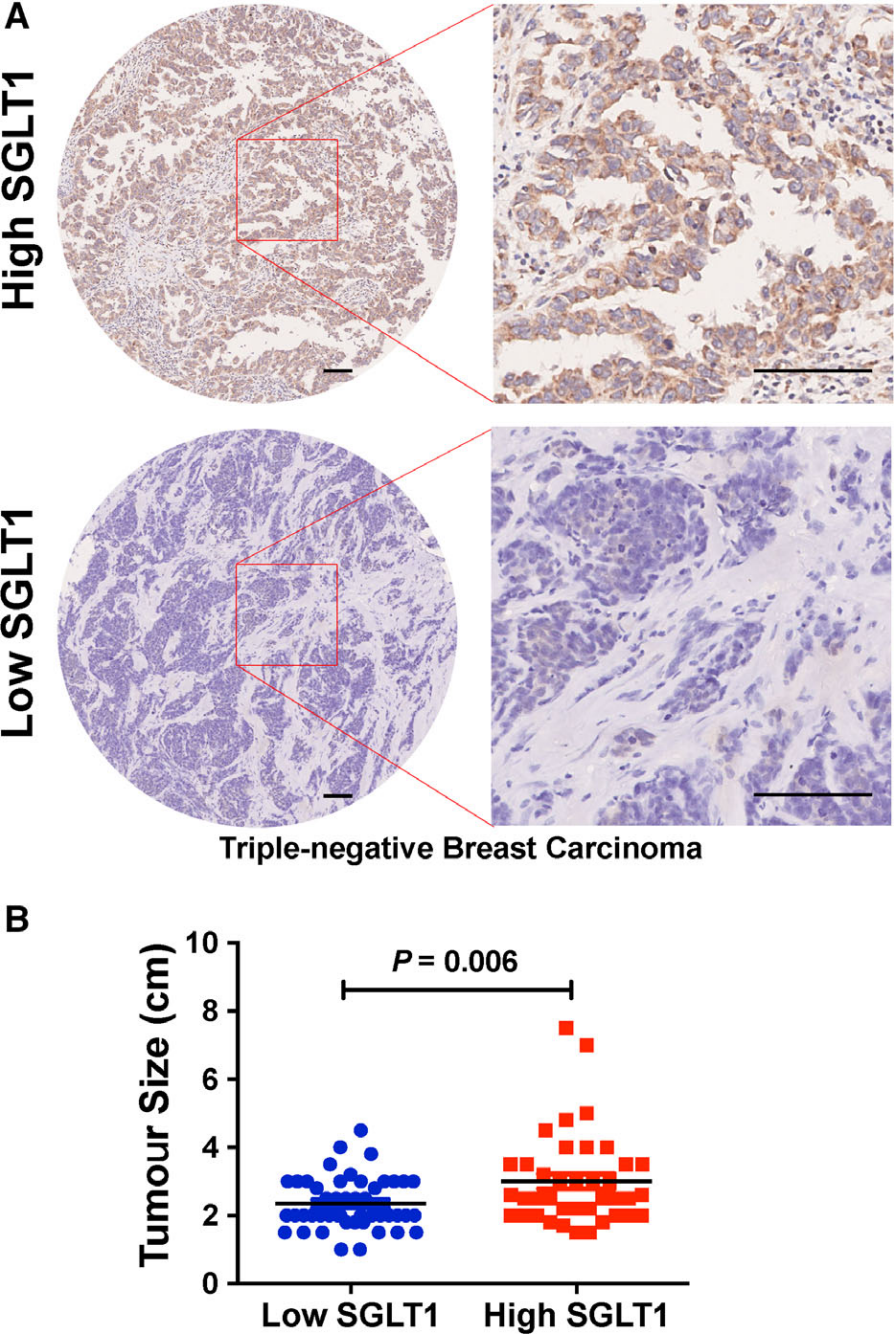
SGLT1 expression levels associate with tumour size in triple‐negative breast carcinoma (TNBC). (A) Representative SGLT1 staining pattern (high or low SGLT1) in 90 TNBC tissue microarray cores. Scale bar: 100 μm. (B) The relationship between SGLT1 expression and tumour size in TNBC samples was analysed. Chi‐square test was performed (*P* = 0.006).

In order to examine whether SGLT1 expression levels correlate with clinical–pathological features in TNBC, we performed immunohistochemistry staining of SGLT1 in TNBC tissue microarrays. Table 1 shows clinical and pathological characteristics of the 90 TNBC patient samples. We found only tumour size was significantly affected by SGLT1 expression (Table 1). Representative images of high and low expression of SGLT1 in TNBC are shown in Fig. 1A. Tumour size was significantly larger in high SGLT1 TNBC samples compared with the low SGLT1 TNBC samples (*P* = 0.006; Fig. 1B).

**Table 1:** The relationship between patients’ clinical–pathological characteristics and SGLT1 expression in TNBC. The other *P* values were obtained by chi‐square test.

Characteristics	*N*	SGLT1		*P* value
		Low expression	High expression	
Age	90			
≤ 50	46	28	18	0.290
> 50	44	21	23	
Location	90			
Left breast	46	23	23	0.406
Right breast	44	26	18	
Grade	90			
I–II	36	19	17	0.832
III	54	30	24	
Size	86			
≤ 2 cm	37	26	11	0.016*
> 2 cm	49	21	28	
Positive LN	35			
≤ 2	21	10	11	0.728^a^
> 2	14	5	9	

a *P* value of Fisher’s exact test. The other *P* values were obtained by chi‐square test. *P < 0.05.

### 3.2 SGLT1 depletion impairs cell viability of TNBC

**Figure 2:**
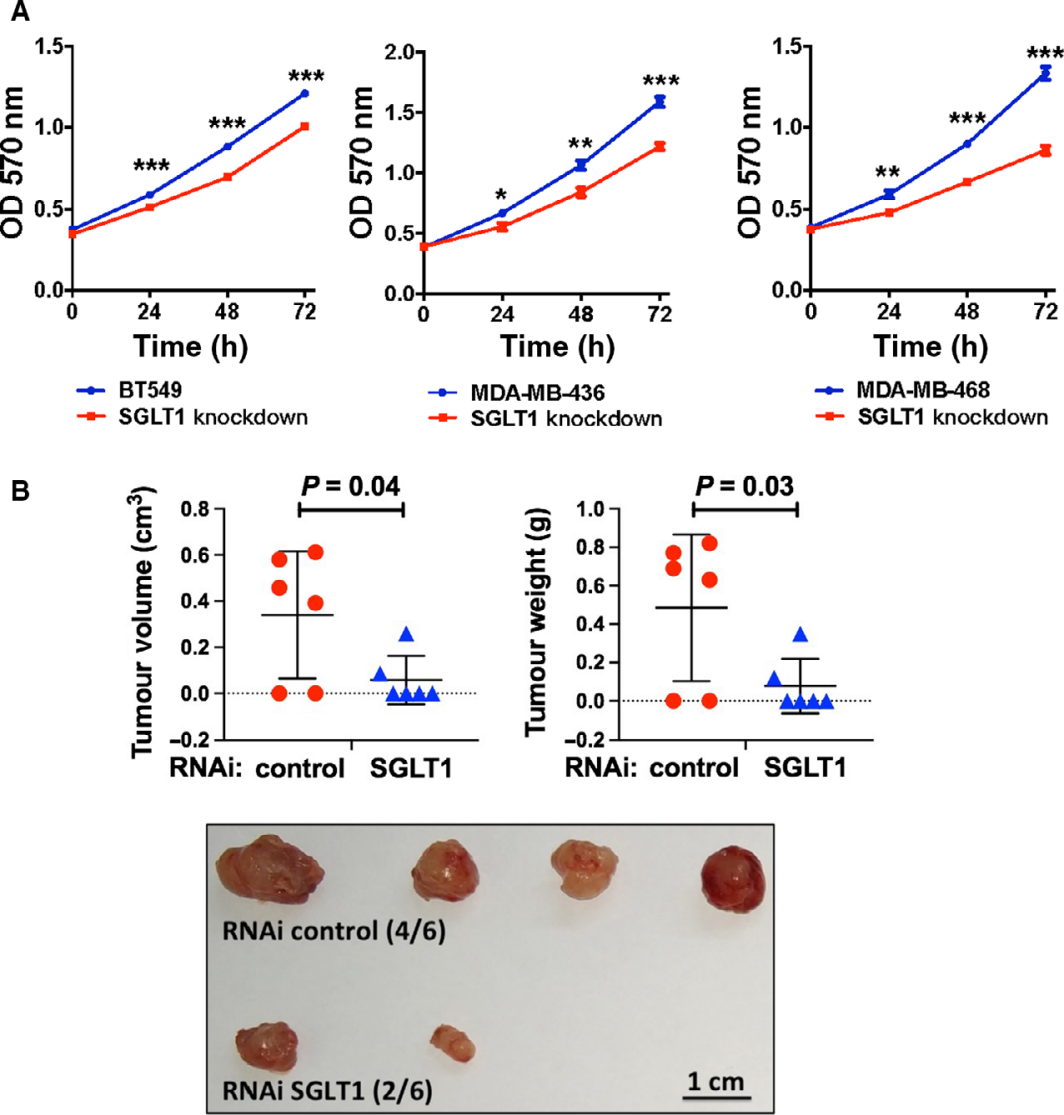
SGLT1 depletion impairs cell viability of TNBC *in vitro* and *in vivo*. (A) Knockdown of SGLT1 in BT549, MDA‐MB‐436 and MDA‐MB‐468 cells resulted in cell growth inhibition as performed by MTT assay. Data are represented as a mean value ± standard deviation of three independent experiments. Student’s *t*‐test was conducted to identify statistical differences in each time point. *P < 0.05, **P < 0.01 and ***P < 0.001. (B) Graphs showing tumour volume (*P* = 0.04) or weight (*P* = 0.03) present in the mice injected with control or SGLT1‐depleted MDA‐MB‐436 cells. Pictures of the tumours formed were also shown. Scale bar: 1 cm. Student’s *t*‐test was performed between control and SGLT1 RNAi groups to identify statistical difference of tumour volume and weight.

Given the fact that SGLT1 levels associate with tumour size, we next checked whether SGLT1 depletion impairs cell viability. MTT assay was performed in control or SGLT1‐depleted MDA‐MB‐436, MDA‐MB‐468 and BT549 cell lines (Fig. S2). The OD values in MDA‐MB‐436, MDA‐MB‐468 and BT549 cells were significantly decreased in SGLT1‐depleted ones compared with the controls in various time points (Fig. 2A). We subsequently performed an *in vivo* study to determine the effect of SGLT1 knockdown on tumour growth. MDA‐MB‐436 tumours were established in the mammary fat pad of female nude mice. As shown in Fig. 2B, tumours were found in four of six mice where control MDA‐MB‐436 cells were injected, whereas only two of six mice had tumours where SGLT1‐depleted MDA‐MB‐436 cells were injected. The volume and weight of tumours present in the mice injected with SGLT1‐depleted MDA‐MB‐436 cells were significantly lower than that in the control group injected with control cells (Fig. 2B). Hence, a reduction in SGLT1 expression is able to inhibit TNBC cell growth *in vitro* and *in vivo*.

### 3.3 TCGA analysis reveals a link between SGLT1 status and AKT signalling in TNBC samples

**Figure 3:**
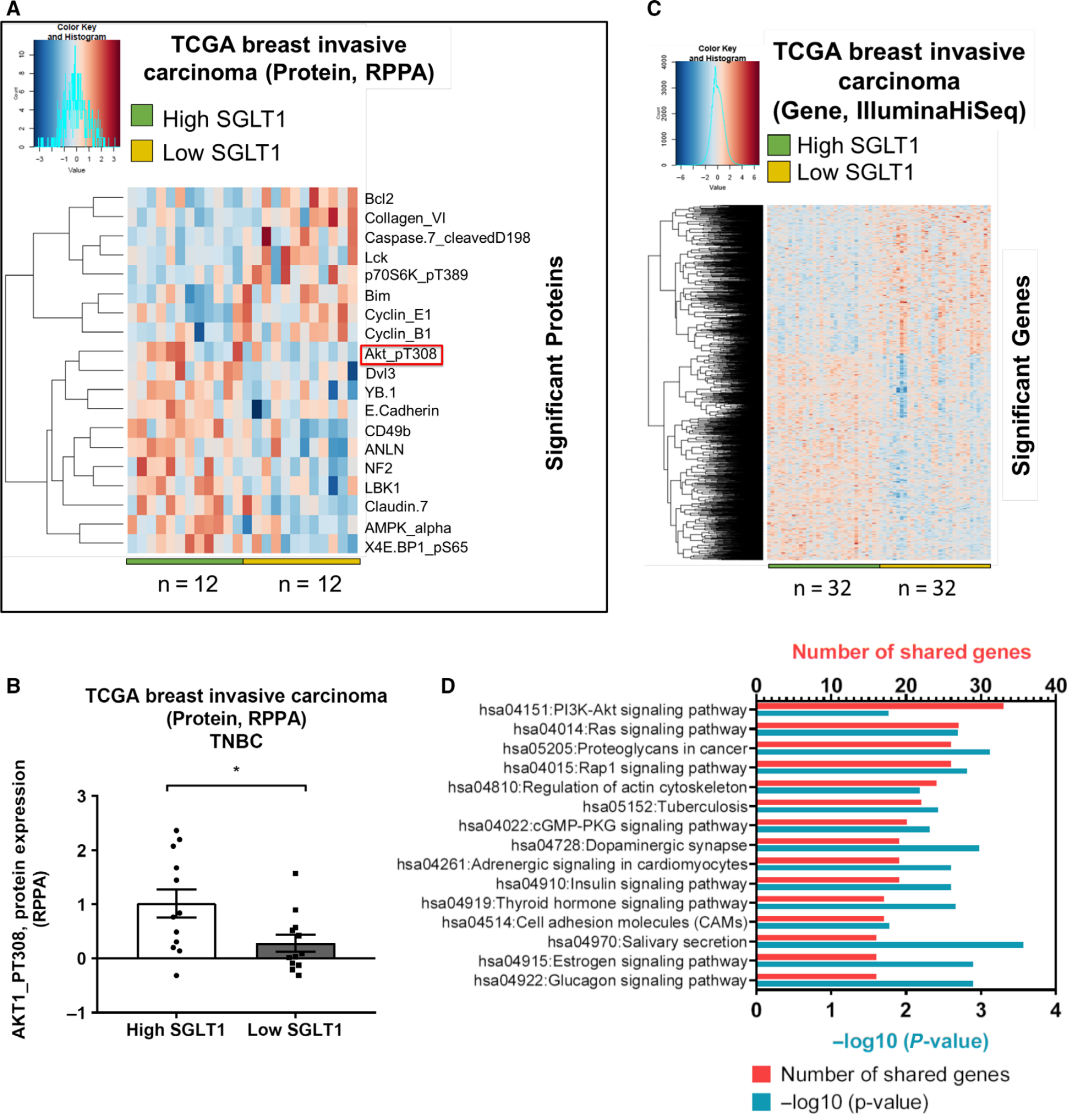
TCGA analysis reveals a link between SGLT1 status and AKT signalling in TNBC samples. Heat maps of the columns indicate each individual sample in high and low SGLT1 groups across each protein (A) and gene (C) in TCGA breast invasive carcinoma data, which obtained from Cancer Genome Browser. Rows indicate protein expressions (RPPA) (A) and gene expressions (IlluminaHiSeq) (C). Dark blue colour indicates low expression of proteins/genes, and dark pink colour illustrates highly expressed proteins/genes. Unpaired *t*‐test was analysed to find the significantly different proteins/genes in TCGA breast invasive carcinoma data sets. *n* represents the number of samples in each group. (B) Box plot shows the protein expression of AKT_pT308 between high and low SGLT1 groups in TNBC, TCGA breast invasive carcinoma. *P < 0.05. (D) The genes that were positively regulated with SGLT1 were analysed in DAVID website to show which pathways are regulated. Histogram shows top 15 pathways based on lowest *P* value. *Y*‐axis shows the pathways, and top *x*‐axis and bottom *x*‐axis show the number of shared genes in each pathway and −log10 (*P* value), respectively.

In order to find how SGLT1 regulates tumour growth, we analysed data from the TCGA project. Protein (RPPA) TCGA breast invasive carcinoma data and Gene, RNASeq (IlluminaHiSeq) TCGA breast invasive carcinoma data sets were obtained from UCSC Cancer Genomics Browser (https://genome-cancer.ucsc.edu/). Protein expression data included 410 breast invasive carcinoma samples and 142 proteins; and gene expression data contained 1215 samples and 20530 genes. Both protein (RPPA) and gene expression, RNASeq (IlluminaHiSeq), breast invasive carcinoma samples from Cancer Genome Browser were aligned with TNBC samples, as shown in Fig. S1. The alignment showed 59 and 160 TNBC samples in protein (RPPA) and gene, RNASeq (IlluminaHiSeq) data, respectively. In both protein and gene expression data sets, the top 20% and bottom 20% samples were separated into two groups: high and low SGLT1‐expressing samples, respectively, and unpaired *t*‐test was performed to find the significantly different proteins/genes between the groups. A heat map of protein expression for 19 proteins, including phosphorylated AKT (AKT_pT308), that were significantly different between high and low SGLT1‐expressing samples (*P* < 0.05) is shown in Fig. 3A. The scatter dot plot showed the protein expression of phosphorylated AKT (AKT_pT308) for each sample in high and low SGLT1 groups (Fig. 3B). A total of 2279 significantly different genes between high and low SGLT1‐expressing samples are shown by the heat map (Fig. 3C).

To demonstrate whether the significantly positive genes with SGLT1 in TCGA data set are involved in the same pathway, DAVID, online website (https://david.ncifcrf.gov) was used to perform a pathway analysis. We found PI3K–AKT signalling pathway is the top pathway that has the highest hit genes, followed by Ras signalling and other pathways (Fig. 3D). These analyses suggested that SGLT1 may interact with AKT signalling pathway.

### 3.4 SGLT1 binds EGFR and positively regulates EGFR activity

**Figure 4:**
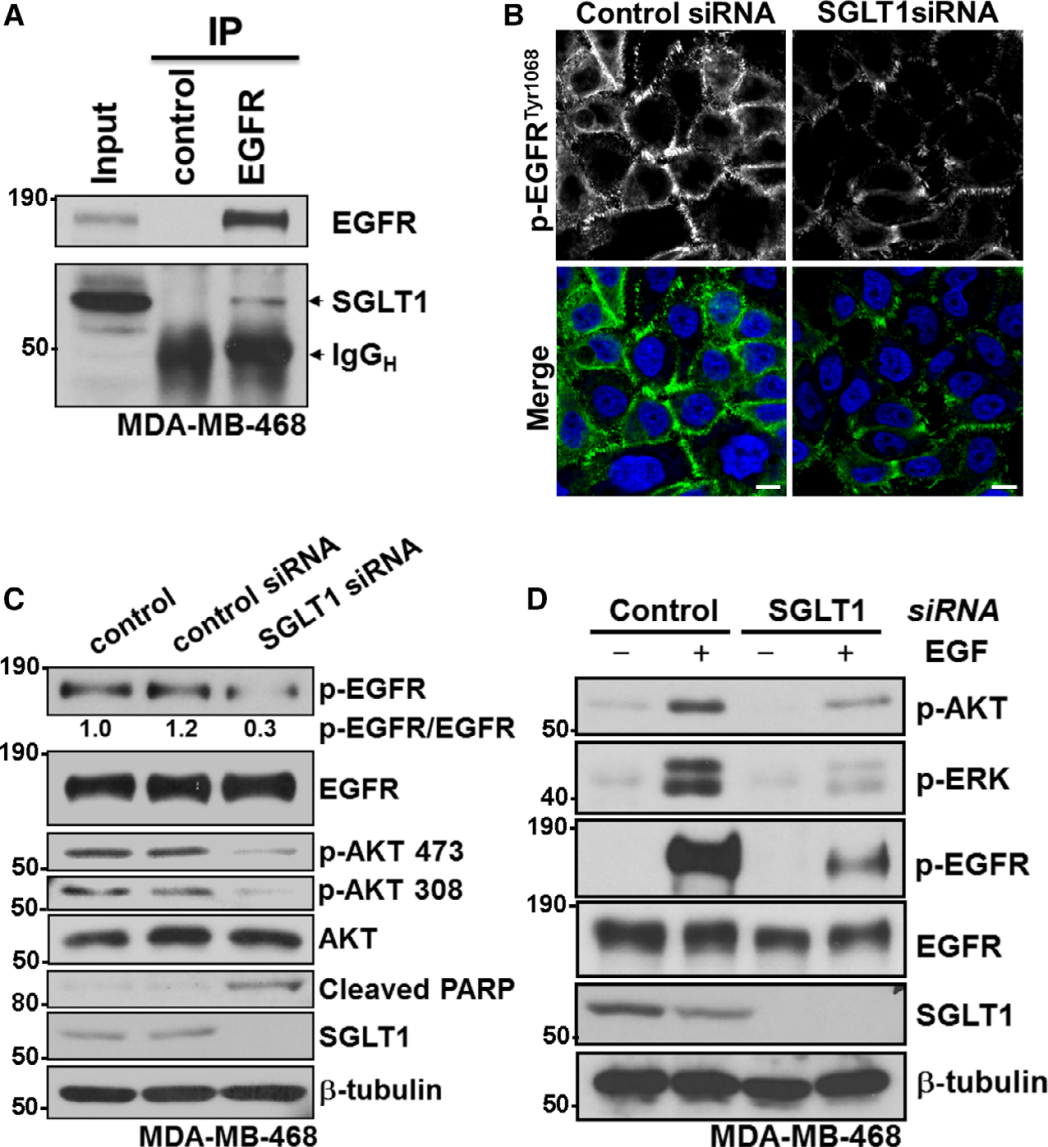
SGLT1 binds EGFR and positively regulates EGFR activity. (A) Total cell lysates from MDA‐MB‐468 cells were immunoprecipitated with an anti‐EGFR antibody or control IgG. EGFR and SGLT1 levels are indicated. IgG_H_ indicates IgG heavy chain. (B) Immunofluorescence staining of Phospho‐EGFR^Tyr1068^ (green) in MDA‐MB‐468 cells transfected with SGLT1 siRNA or control siRNA. TO‐PRO‐3 (blue) was used to stain nuclei. Scale bar: 10 µm. (C) Protein expression of Phospho‐EGFR^Tyr1068^, EGFR, Phospho‐AKT Ser473, Phospho‐AKT Ser308, AKT, Cleaved PARP Asp214 and SGLT1 in MDA‐MB‐468 cells with indicated treatments. β‐tubulin was used as a loading control. (D) Protein expression of Phospho‐EGFR^Tyr1068^, EGFR, Phospho‐AKT Ser473, Phospho‐ERK (Thr202/Tyr204) and SGLT1 in MDA‐MB‐468 cells with indicated treatments. β‐tubulin was used as a loading control.

Search Tool for the Retrieval of Interacting Genes (https://string-db.org) analysis showed the interaction between SGLT1 and EGFR (Fig. S3), which was reported earlier (Ren *et al.*, 2013; Weihua *et al.*, 2008). In fact, we also observed EGFR–SGLT1 interaction in MDA‐MB‐468 human breast cancer cells. Endogenous EGFR and SGLT1 co‐immunoprecipitated together in MDA‐MB‐468 cells (Fig. 4A).

Given that SGLT1 interacts primarily with the auto‐phosphorylation domain of EGFR (Ren *et al.*, 2013), we asked whether SGLT1 status could also affect EGFR activity on the other hand. Consistently, the immunofluorescence signal of phospho‐EGFR^Tyr1068^ was at the cell membrane in MDA‐MB‐468 cells (Fig. 4B). To test the biological importance of the observed EGFR–SGLT1 interaction, we examined the level of phospho‐EGFR^Tyr1068^ by immunofluorescence staining in MDA‐MB‐468 cells treated with control small interfering RNA (siRNA) or siRNA against SGLT1. Importantly, SGLT1 depletion by RNA interference (RNAi) largely abolished the membrane signal of phospho‐EGFR^Tyr1068^ (Fig. 4B). For most of the analyses described here, we used pools of four siRNA against each gene (‘SMARTpools’ from Dharmacon). However, deconvolution of siRNA pools into their constituent individual oligonucleotides is an important step in minimizing the potential for off‐target effects to compromise the analysis of gene knockdown studies (Echeverri *et al.*, 2006). Figure S4 showed that deconvolution of SGLT1 siRNA SMARTpool, two out of four different oligonucleotides, clearly led to the downregulation of SGLT1 and reduced level of phospho‐EGFR^Tyr1068^.

We next asked whether SGLT1 status could affect the activity of EGFR downstream pathways, which was investigated by examining the EGFR signalling cascade in MDA‐MB‐468 cells transfected with control siRNA or SGLT1 siRNA. The basal phosphorylation levels of EGFR (Tyr1068) and AKT (Ser473 and Thr308) were reduced in SGLT1‐depleted MDA‐MB‐468 cells compared with control cells or control siRNA‐treated cells. Total protein levels of EGFR and AKT, however, were not affected (Fig. 4C). Following SGLT1 gene silencing in MDA‐MB‐468 cells, a rapid and profound loss of cell viability was observed, most likely resulting from a robust induction of apoptosis. This is evident by the detection of poly ADP ribose polymerase (PARP) cleavage, a well‐described indicator of effector caspase activation and consequent cell death (Fig. 4C).

The difference was more notable upon epidermal growth factor (EGF) treatment. A much stronger response induced by addition of EGF was observed in control siRNA‐treated cells compared with SGLT1‐depleted MDA‐MB‐468 cells, reflected by the phosphorylation levels of EGFR (Tyr1068), ERK (Thr202/Tyr204) and AKT (Ser473; Fig. 4D). These data suggest that by binding to EGFR, SGLT1 potentiates EGFR signalling.

**Figure 5:**
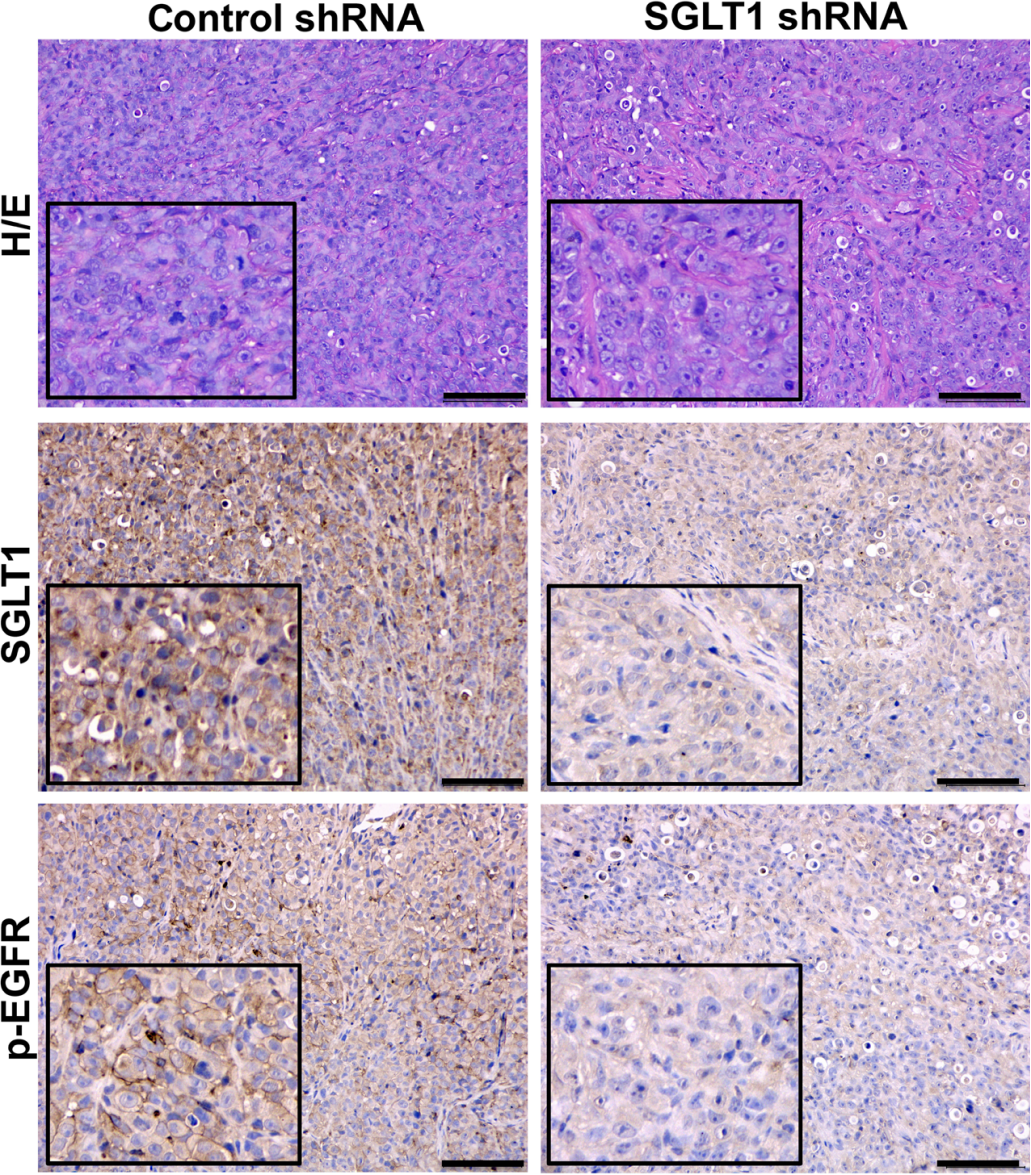
Downregulation of SGLT1 reduces EGFR phosphorylation level *in vivo*. Adjacent tumour sections from representative cases of tumour xenografts formed by MDA‐MB‐436 cells treated with control or SGLT1 shRNA were stained with H/E. The expression of SGLT1 and p‐EGFR is also indicated. Scale bars: 100 μm.

**Figure 6:**
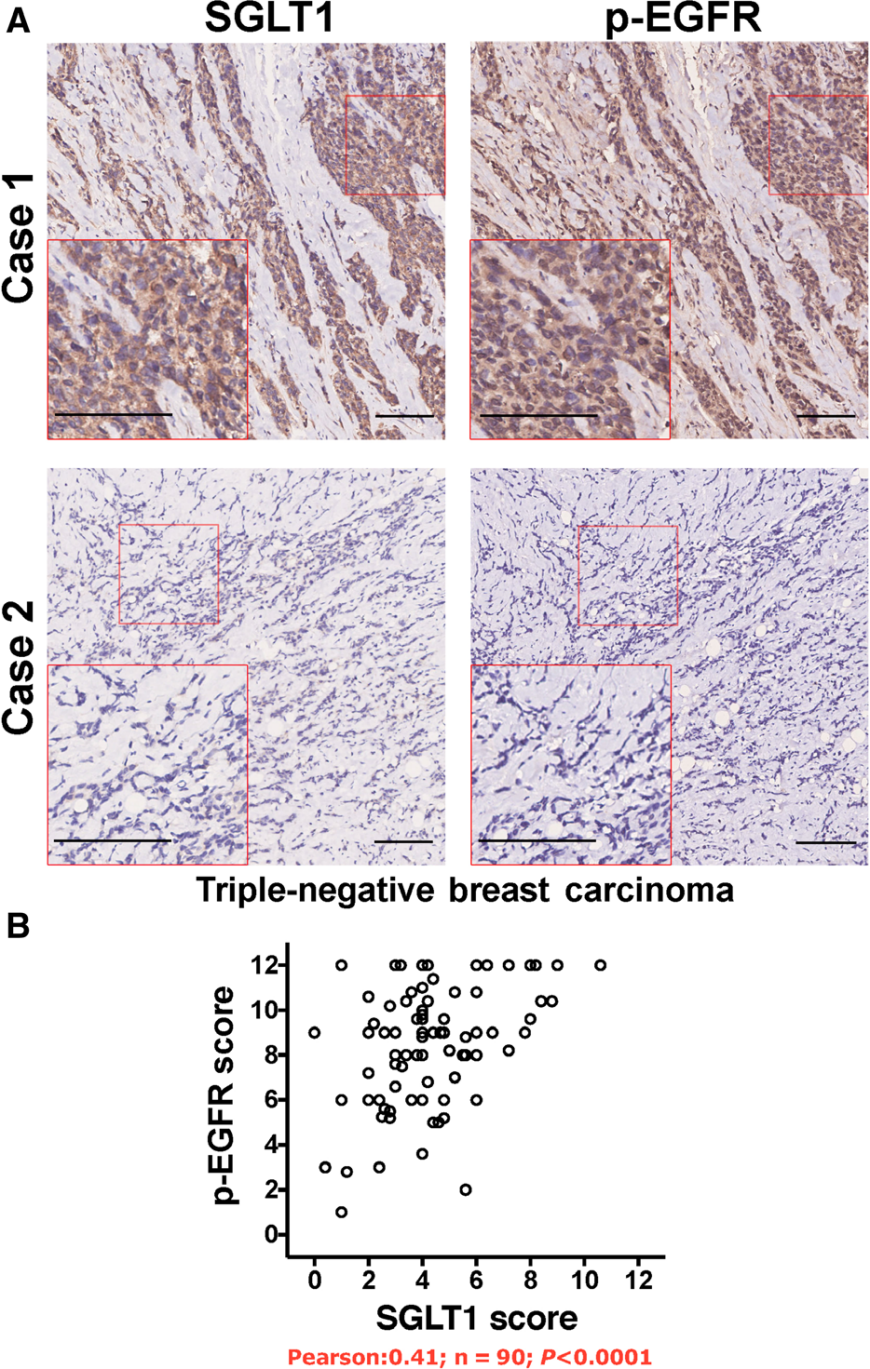
SGLT1 expression levels positively correlate with EGFR phosphorylation in TNBC. (A) Adjacent tumour sections from representative cases show SGLT1 and p‐EGFR expression in TNBC. Scale bar: 100 μm. (B) The relationship between SGLT1 expression and EGFR phosphorylation was analysed by Pearson correlation test (*R* = 0.41, *n* = 90, *P* < 0.0001).

To further validate the *in vitro* findings, the regulation of EGFR phosphorylation by SGLT1 was investigated *in vivo* using tumour sections derived from control shRNA or SGLT1 shRNA‐transfected MDA‐MB‐436 cells (Fig. 2B). Using adjacent tumour sections derived from MDA‐MB‐436–control shRNA cells, we observed positive SGLT1 and EGFR phosphorylation. In tumours derived from MDA‐MB‐436–SGLT1 shRNA cells, SGLT1‐negative tumour regions showed reduced levels of EGFR phosphorylation (Fig. 5). In addition, the correlation of SGLT1 expression and EGFR phosphorylation levels was analysed in TNBC samples (Fig. 6). High SGLT1 correlated with high EGFR phosphorylation levels in TNBC (*P* < 0.0001). These observations agree with the finding that SGLT1 potentiates EGFR signalling in TNBC.

## 4 Discussion

Chemotherapy has been the only systemic treatment option for TNBCs for several years to increase the overall survival rate of the patients (Khosravi‐Shahi *et al.*, 2018), but chemotherapy resistance is the major challenge for the treatment of the patients with TNBC (Lee and Djamgoz, 2018). Therefore, several studies detected the molecular changes before and after the chemotherapy to identify potential targeted therapy for TNBC (Balko *et al.*, 2014). Identification of targeted therapy for the patients with TNBC could help to prevent the risk of resistance of the treatment option and recurrence of the disease. In this study, we showed that targeting of SGLT1 has the potential to treat TNBC. We found SGLT1 status associates with tumour size in TNBC. Similarly, SGLT1 expression showed significant association with clinical–pathological characteristics and prognosis of ovarian cancer; SGLT1 overexpression significantly correlated with increased pT status and poor prognosis (Lai *et al.*, 2012). Guo *et al. *(2011) also reported that higher expression of SGLT1 significantly associated with the clinical stage of colorectal cancer (Guo *et al.*, 2011).

SGLT1 is an active glucose transporter and plays a critical role in glucose absorption and retention in the body (Wright *et al.*, 2011). In cancer cells, a high rate of glucose uptake is required to meet the increased energy needs and leads to abnormal growth of cancerous cells (Hanahan and Weinberg, 2011). Depletion or inhibition of SGLT1 may reduce the energy supply to the cancer cells. In addition, we demonstrated that downregulation of SGLT1 dampened EGFR‐AKT or EGFR‐ERK pathways activity, which phosphorylates a plethora of targets to activate the cell cycle, prevent apoptosis and trigger cellular growth (Downward, 2003; Manning and Cantley, 2007). As a result, SGLT1 depletion induced apoptosis in TNBC cells and inhibited their growth *in vitro* and *in vivo*. Consistently, overexpression of SGLT1 protected renal epithelial cells (Ikari *et al.*, 2005) and intestinal epithelial cells (Yu *et al.*, 2008) from apoptosis.

Upon activation by its growth factor ligands, EGFR undergoes a transition from an inactive monomeric form to an active homodimer (Yarden and Schlessinger, 1987). EGFR dimerization stimulates its intrinsic intracellular protein–tyrosine kinase activity. As a result, autophosphorylation of several tyrosine residues in the C‐terminal domain of EGFR occurs (Downward *et al.*, 1984; Lemmon *et al.*, 2014). This autophosphorylation elicits downstream activation and signalling. For example, growth factor receptor‐bound protein 2 binds activated EGFR at phospho‐Tyr1068, which is crucial to the EGF‐induced activation of Ras signalling pathway (Rojas *et al.*, 1996). It was demonstrated autophosphorylation of EGFR is taking place primarily by activated EGFR located at the cell membrane (Sousa *et al.*, 2012). Downregulation of SGLT1 by RNAi resulted in the decreased level of phospho‐EGFR^Tyr1068^, and as a result, the activity of downstream signalling pathways (such as AKT and ERK) was inhibited, suggesting that SGLT1 may facilitate the autophosphorylation of EGFR upon ligand binding. This result was also supported by significantly positive correlation between phospho‐EGFR and SGLT1 in TNBC samples.

In line with the previous report indicating that loss of EGFR protein but not its tyrosine kinase activity sensitized cancer cells to chemotherapeutic agent (Weihua *et al.*, 2008), EGFR tyrosine kinase inhibitors did not produce therapeutic effects for certain cancers (Cohen *et al.*, 2003; Dancey and Freidlin, 2003; Fukuoka *et al.*, 2003). It has been highlighted EGFR tyrosine kinase inhibitors or monoclonal antibodies against TNBC showed low response rate, and the combination treatment with tyrosine kinase inhibitors or monoclonal antibodies and chemotherapy also showed low response rate and no benefits in the survival rate of patients (Baselga *et al.*, 2013; Carey *et al.*, 2012; Finn *et al.*, 2009; Lee and Djamgoz, 2018; Schuler *et al.*, 2010). Therefore, further works are needed to find out the best way to use EGFR targeted therapy in TNBC. EGFR–SGLT1 interaction stabilizes SGLT1 for the high uptake of glucose by cancerous cells and leads to the progression of cancer (Ren *et al.*, 2013; Weihua *et al.*, 2008). EGFR depletion (Weihua *et al.*, 2008) or deletion of the SGLT1 interacting domain in EGFR promoted the downregulation of SGTL1 via the proteasome machinery (Ren *et al.*, 2013). Together, targeting SGLT1 itself or EGFR–SGLT1 interaction might potentially provide novel therapeutics for TNBC patients.

## 5 Conclusion

In summary, this study shows that high levels of SGLT1 are associated with greater tumour size in TNBC. SGLT1 depletion compromises cell growth *in vitro* and *in vivo*. We further demonstrate that downregulation of SGLT1 results in decreased levels of phospho‐EGFR, and as a result, the activity of downstream signalling pathways (such as AKT and ERK) is inhibited. Hence, SGLT1 is required for the survival of TNBC via potentiation of EGFR activity.

## Conflict of interest

The authors declare no conflict of interest.

## Author contributions

HL, AE and PP contributed equally in this work.

## Supporting information


**Fig. S1**. TCGA analysis of SGLT1 expression levels in different molecular subtypes of breast invasive carcinoma samples (TCGA, Provisional).

**Fig. S2**. Knockdown of SGLT1 in TNBC cells via RNAi.

**Fig. S3**. SGLT1 and its interacting partners.

**Fig. S4**. SGLT1 positively regulates EGFR activity.
