# Supplementary figures and images for: SGLT1 is required for the survival of triple‐negative breast cancer cells via potentiation of EGFR activity
Source: Mol Oncol. 2019 Jun 14;13(9):1874–86. doi: 10.1002/1878-0261.12530 (PMC6717760; doi:10.1002/1878-0261.12530)

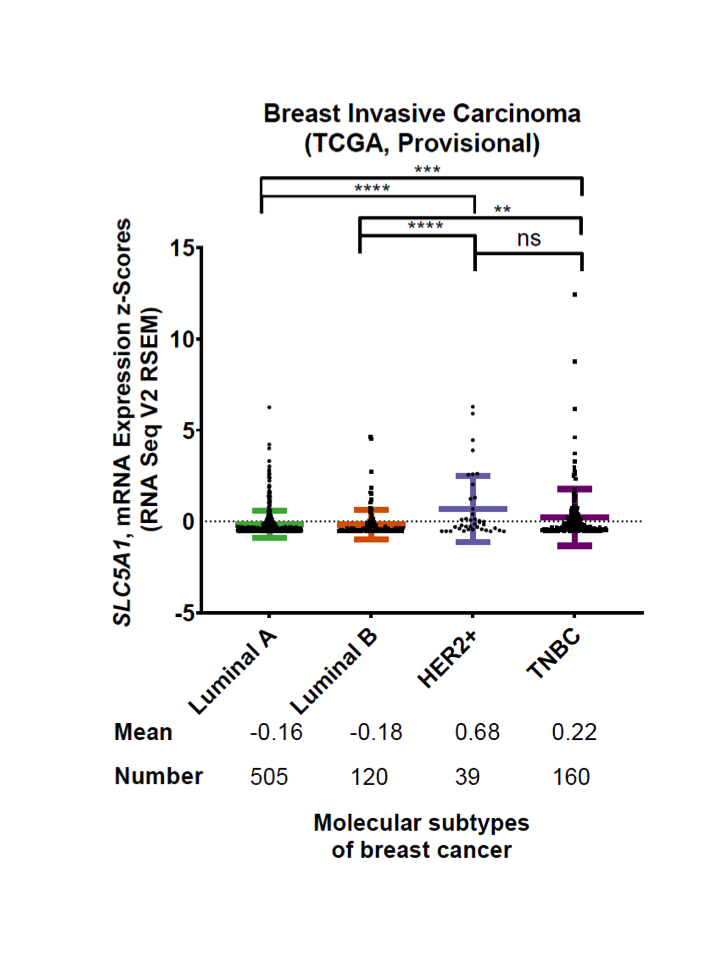

Supplement: Supplementary file 1 — Fig. S1. TCGA analysis of SGLT1 expression levels in different molecular subtypes of breast invasive carcinoma samples (TCGA, Provisional). [file MOL2-13-1874-s001.tiff]

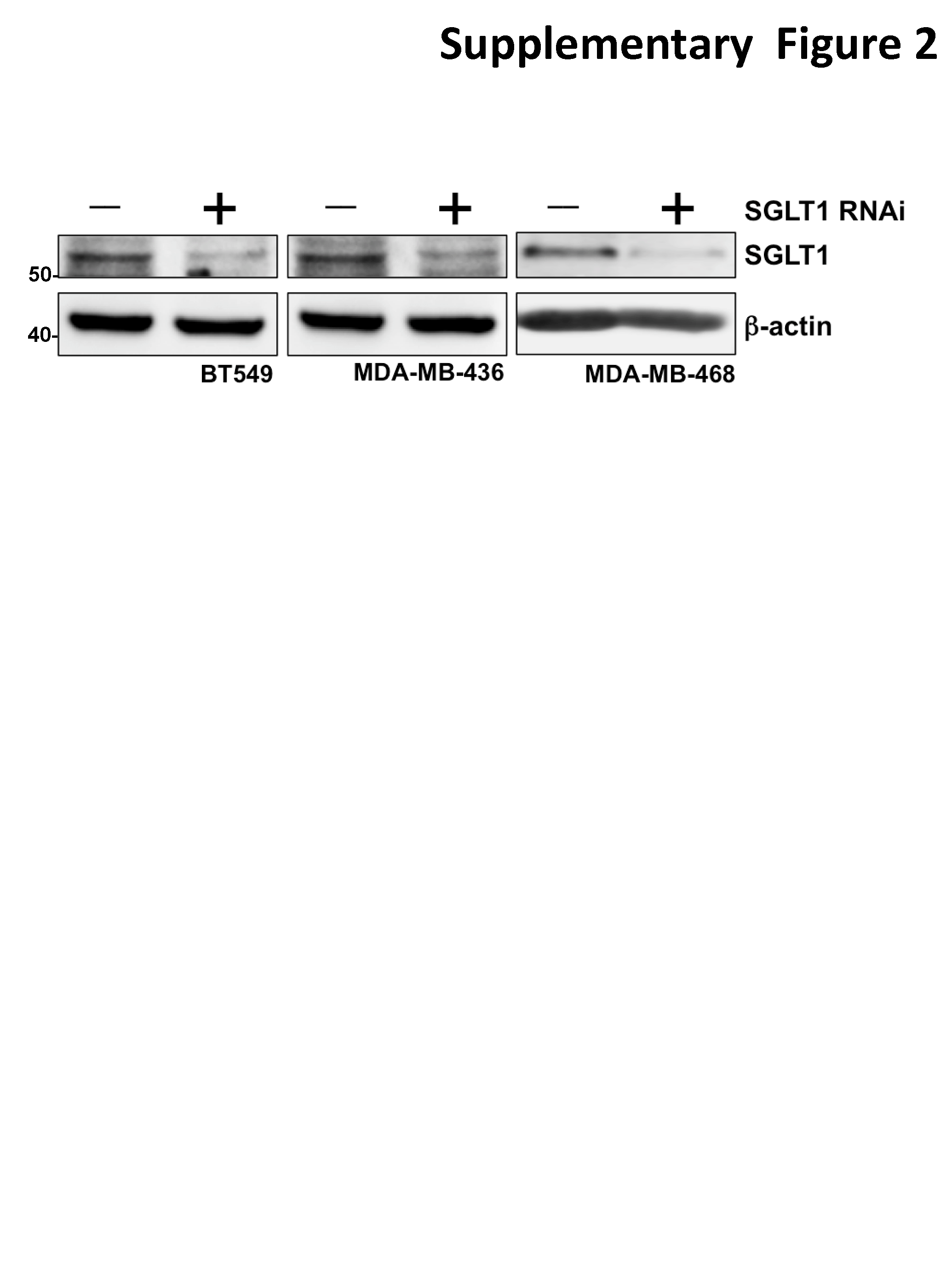

Supplement: Supplementary file 2 — Fig. S2. Knockdown of SGLT1 in TNBC cells via RNAi. [file MOL2-13-1874-s002.tiff]

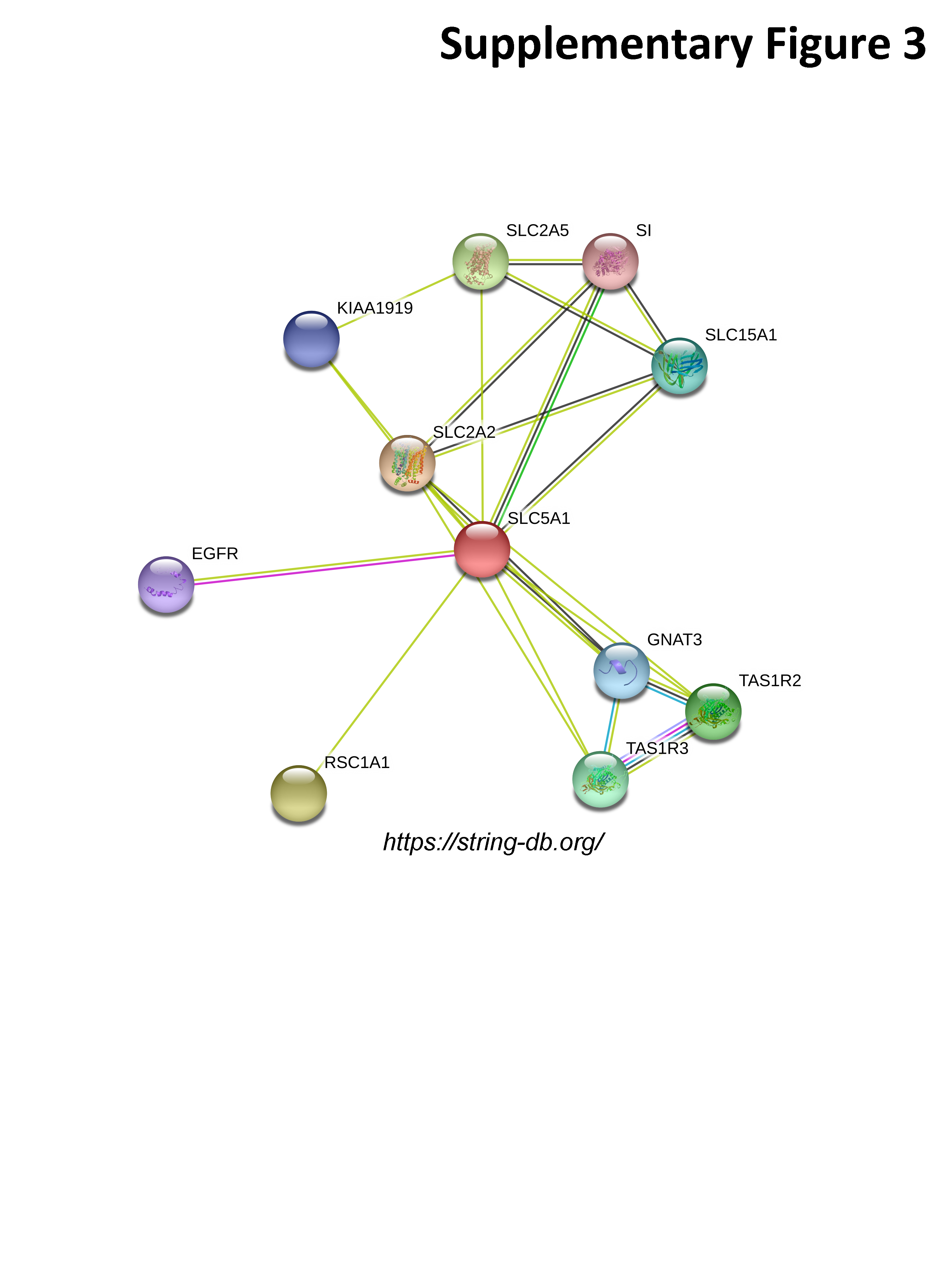

Supplement: Supplementary file 3 — Fig. S3. SGLT1 and its interacting partners. [file MOL2-13-1874-s003.tiff]

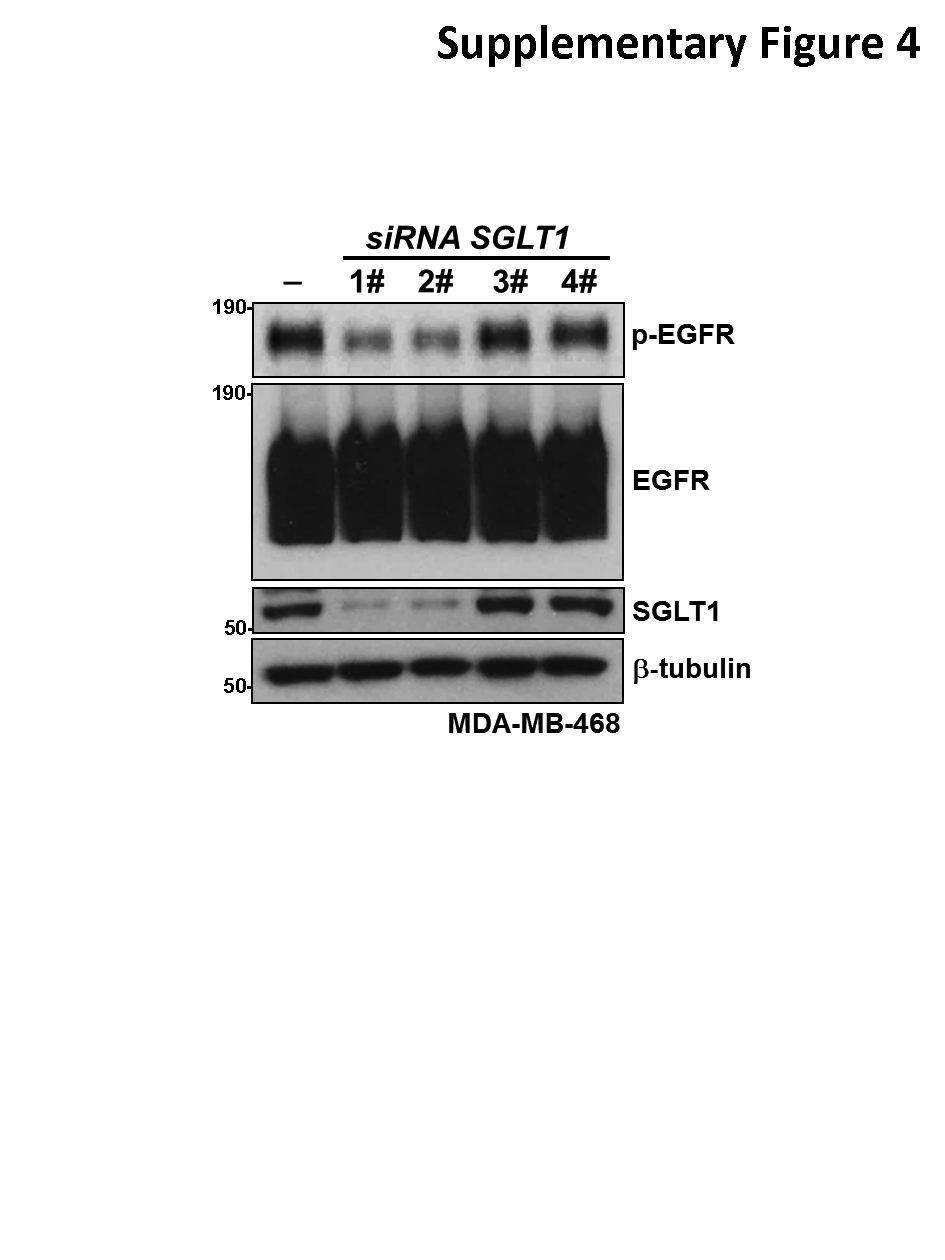

Supplement: Supplementary file 4 — Fig. S4. SGLT1 positively regulates EGFR activity. [file MOL2-13-1874-s004.tiff]
