# Supplementary material for: SGLT1 is required for the survival of triple‐negative breast cancer cells via potentiation of EGFR activity
Source: Mol Oncol. 2019 Jun 14;13(9):1874–86. doi: 10.1002/1878-0261.12530 (PMC6717760; doi:10.1002/1878-0261.12530)
Supplement: Supplementary file 5 [file MOL2-13-1874-s005.docx]

**SGLT1 is required for the survival of triple negative breast cancer cells via potentiation of EGFR activity**

***Supplementary Figure Legends***

**Supplementary Figure 1. TCGA analysis of SGLT1 expression levels in different molecular subtypes of breast invasive carcinoma samples (TCGA, Provisional).** SGLT1 mRNA expression in four different molecular subtypes of breast cancer. The average of all samples in each molecular subtypes of breast cancers were calculated. Error bars indicate mean ± standard deviation (SD). One-way ANOVA was performed for statistical analysis. ns *P* ≥ 0.05; ***P* ≤ 0.01; ****P* ≤ 0.001 and *****P* ≤ 0.0001.

**Supplementary Figure 2. Knockdown of SGLT1 in TNBC cells via RNAi.** Protein expression of SGLT1 in BT549, MDA-MB-436 and MDA-MB-468 cells with the indicated treatments. β-actin was used as a loading control.

**Supplementary Figure 3. SGLT1 and its interacting partners.** STRING (Search Tool for the Retrieval of Interacting Genes) (<https://string-db.org/>) analysis showed the interaction between SGLT1 and EGFR.

**Supplementary Figure 4. SGLT1 positively regulates EGFR activity.** Protein expression of Phospho-EGFR^Tyr1068^, EGFR and SGLT1 in MDA-MB-468 cells with indicated treatments. β-tubulin was used as a loading control.
